# Towards transforming the mental health services of the Philippines

**DOI:** 10.1016/j.lanwpc.2023.100935

**Published:** 2023-10-09

**Authors:** Rowalt Alibudbud

**Affiliations:** Department of Sociology and Behavioral Sciences, College of Liberal Arts, De La Salle University, Manila, Philippines

The WHO Special Initiative for Mental Health, aiming to establish accessible and affordable mental healthcare, reported insights into the challenges faced by participating countries, including the Philippines.^1^ These challenges encompass service delivery, governance, workforce, financing, and stigma.^1^ In the Philippines, the prevalence of mental disorders ranged between 11.3% and 11.6%, with an average annual increase of 2.0%; increasing from 7.0 to 12.5 million Filipinos diagnosed with a mental disorder between 1990 and 2019.^2^ Anxiety and depression are the prevailing conditions.^2^ Considering these challenges, WHO advocated a strategic approach comprising increasing political prioritization and funding, developing a sustainable and transformative model of care, and promoting a collaborative approach to practical context-specific measures.^1^

The enactment of the Philippine Mental Health Act in 2018 signaled a step towards a comprehensive mental health framework.^2^^,^^3^ Historically, mental health financing was low, accounting for only 5% of the healthcare budget.^2^^,^^3^ Nevertheless, developments in Philippine mental healthcare are promising, including the nearly twenty-fold increase in government mental health financing from 57 million to 1 billion between 2022 and 2023.^1^ Moreover, The Philippines is also piloting a mental health package for outpatient settings.^1^ However, timely payment of claims is essential, as reports indicate that reimbursements of expenditures can be delayed for up to three months.^4^ Therefore, notwithstanding the improvement in mental health financing, it is imperative to explore sustainable funding sources, including private sector participation.

WHO also introduced the Consolidated Framework for Implementation Research.^1^ This framework systematically assesses the factors influencing mental healthcare accessibility and affordability, which include intervention, internal and external settings, individuals, and the process.^1^ Challenges such as focus on tertiary-level services and the complexity of primary mental health care requirements are observed in the Philippines. Mental health services in the country are concentrated in psychiatric hospitals and outpatient care services in general hospitals, with limited integration into primary healthcare and informal community support.^2^ Therefore, interventions should prioritize the establishment of community-based mental health services, and processes to improve mental healthcare accessibility and affordability.^1^ It is also pivotal to advocate for integrating different levels of care to ensure an efficient and responsive mental healthcare system. There are approximately 1600 psychologists and 500 psychiatrists in the country, serving about 110 million Filipinos.^2^^,^^5^ Resources in tertiary-level services, including psychiatrists and emergent services, can be harnessed to facilitate the training of primary care workers and establish referral centers within communities to cater to individuals in need of specialized care.

In the Philippines, the internal setting of the WHO Framework can refer to the partnership of WHO, the Department of Health, and the Philippine Council for Mental Health (PCMH), an agency entrusted with formulating mental health policies and services.^6^ To strengthen this partnership, an array of experts and stakeholders is essential. Leading mental health researchers can be included in PCMH to more effectively contextualize the mental health system, considering the limited mental health research available in the country necessary to inform stakeholders. For instance, Scimago reveals 431 publications within the psychiatry and mental health category from the Philippines between 1996 and 2022.^7^ This number pales compared to other countries with similar economies and regions (i.e., Vietnam, Thailand).^7^

The external setting of the WHO framework encompasses the complex environment influencing interventions. Filipino culture offers valuable resources to enhance Filipinos' mental well-being and social support, encompassing tight-knit community structures and Filipino psychology concepts, such as *kapwa*.^8^^,^^9^ However, stigma rooted in Filipino culture remains a challenge, with some attributing mental disorders to personal weaknesses or supernatural causes.^8^ As a result, mental disorders are seen as socially unacceptable, leading Filipinos to turn to friends and family for assistance and avoid professional mental healthcare.^2^^,^^8^ In the Philippines, advocates have pushed for the Mental Health Act implementation, leading to programs within communities and sectors.^1^ For example, school-based programs have been introduced over the years.^1^ Nevertheless, enhancing transparency, accountability, support, and incentives is necessary to ensure the effective implementation of these interventions.^1^ Therefore, it becomes essential to acknowledge and incentivize the contributions of key actors who advance the understanding and transform the Philippine mental health system. For example, annual recognitions can incentivize key actors (i.e., researchers and community-based programs) to further promote mental health system transformation.

Ensuring the quality of personnel who lead community-based interventions in mental health system development is vital.^1^ However, corruption and nepotism within the Philippines have posed historical and contemporary challenges.^10^ Strategies to mitigate these challenges could include implementing transparent and well-defined scoring systems beyond conventional qualifications for prospective administrators and experts. For instance, the assessment of experts can encompass competency considerations, such as publications and h-indices, together with non-academic metrics like professional memberships and clinical experience.

Multi-stakeholder participatory engagement process is crucial for mental health system transformation in the Philippines.^1^ Stakeholders' involvement in developing and evaluating outcome indicators can help ensure that key actors and stakeholders, including service users and providers, caregivers, administrators, and researchers, are considered. Overall, the increase in mental health prioritization in the Philippines is promising. However, strengthening current developments is essential for transforming mental health services. Efficient resource utilization is critical to a sustainable model of care. Lastly, a shared approach to contextualized interventions, along with informed stakeholders, is vital to transforming Philippine mental healthcare.

## Declaration of interests

RA has previously received funding from the WHO and UNICEF for mental health projects within the Philippines.
